# Attention-Based DSC-ConvLSTM for Multiclass Motor Imagery Classification

**DOI:** 10.1155/2022/8187009

**Published:** 2022-05-05

**Authors:** Li Li, Nan Sun

**Affiliations:** State Key Laboratory of Networking and Switching Technology, Beijing Laboratory of Advanced Information Networks, Beijing University of Posts and Telecommunications, Beijing, China

## Abstract

With the rapid development of deep learning, researchers have gradually applied it to motor imagery brain computer interface (MI-BCI) and initially demonstrated its advantages over traditional machine learning. However, its application still faces many challenges, and the recognition rate of electroencephalogram (EEG) is still the bottleneck restricting the development of MI-BCI. In order to improve the accuracy of EEG classification, a DSC-ConvLSTM model based on the attention mechanism is proposed for the multi-classification of motor imagery EEG signals. To address the problem of the small sample size of well-labeled and accurate EEG data, the preprocessing uses sliding windows for data augmentation, and the average prediction loss of each sliding window is used as the final prediction loss for that trial. This not only increases the training sample size and is beneficial to train complex neural network models, but also the network no longer extracts the global features of the whole trial so as to avoid learning the difference features among trials, which can effectively eliminate the influence of individual specificity. In the aspect of feature extraction and classification, the overall network structure is designed according to the characteristics of the EEG signals in this paper. Firstly, depth separable convolution (DSC) is used to extract spatial features of EEG signals. On the one hand, this reduces the number of parameters and improves the response speed of the system. On the other hand, the network structure we designed is more conducive to extract directly the direct extraction of spatial features of EEG signals. Secondly, the internal structure of the Long Short-Term Memory (LSTM) unit is improved by using convolution and attention mechanism, and a novel bidirectional convolution LSTM (ConvLSTM) structure is proposed by comparing the effects of embedding convolution and attention mechanism in the input and different gates, respectively. In the ConvLSTM module, the convolutional structure is only introduced into the input-to-state transition, while the gates still remain the original fully connected mechanism, and the attention mechanism is introduced into the input to further improve the overall decoding performance of the model. This bidirectional ConvLSTM extracts the time-domain features of EEG signals and integrates the feature extraction capability of the CNN and the sequence processing capability of LSTM. The experimental results show that the average classification accuracy of the model reaches 73.7% and 92.6% on two datasets, BCI Competition IV Dataset 2a and High Gamma Dataset, respectively, which proves the robustness and effectiveness of the model we proposed. It can be seen that the model in this paper can deeply excavate significant EEG features from the original EEG signals, show good performance in different subjects and different datasets, and improve the influence of individual variability on the classification performance, which is of practical significance for promoting the development of brain-computer interface technology towards a practical and marketable direction.

## 1. Introduction

Brain-computer interface (BCI) technology is a frontier research direction of multidisciplinary integration. It establishes a direct communication pathway between the human brain and general-purpose/specialized computing equipment by means of electroencephalogram (EEG) signals acquisition and decoding technology so as to interact with the outside world [1]. BCI is primarily used in medical treatment for severely ill patients with neurological or muscular disabilities. With the continuous progress of science and technology, the application of the brain-computer interface has been extended to many industries such as education, health care, and entertainment. Brain-computer interface technology is centered on human thinking and ideas, analyzes EEG signals through signal processing algorithm to obtain corresponding control instructions, and finally realizes the control of terminal devices. The control signals of BCI can be a variety of brain functional signals. Among them, the BCI system based on EEG is considered to be the most practical BCI system in the future due to its advantages in ease of use, safety, and system cost. The principle of motor imagery (MI) BCI system is an event-related desynchronization/synchronization phenomenon of EEG signals generated during motor imagery. The generation of Motor Imagery EEG (MI-EEG) signals does not depend on external stimuli and only requires the subjects to imagine the corresponding action. The system design is relatively simple, the experiment is relatively convenient, and the signal transmission rate is fast. It has become one of the main research directions in the field of brain-computer interface. It is the focus and difficulty of relevant brain-computer interface technology to realize the accurate recognition of MI-EEG for outputting control instructions or providing feedback to users.

It is extremely challenging to extract stable and valuable features from motor imagery EEG signals. The traditional EEG feature extraction algorithms mainly include time domain method, frequency domain method, time frequency method, and spatial filtering method. Time-domain method includes power spectrum analysis and phase-locked value [2], frequency-domain method includes autoregression model [3] and digital filter [4], time-frequency method includes wavelet transform [5] and wavelet packet transform [6], and spatial filtering method is mainly common spatial pattern (CSP) algorithm. In current studies, CSP has been proved to be the most effective and widely used among traditional methods. Many studies are based on the extension of the CSP method to improve the classification accuracy of MI tasks. Reference [7] first applied CSP to the classification of motor imagery EEG signals. However, the features extracted using CSP lack frequency domain information. Reference [8] proposed filter bank common spatial pattern (FBCSP), which incorporates frequency domain information by the specific filter for frequency domain selection followed by CSP feature extraction.

EEG signal classification is the most critical step for BCI systems. The traditional classification algorithms mainly include linear discriminant analysis (LDA), support vector machine (SVM), common spatial pattern (CSP), and artificial neural network (ANN). In the emerging deep learning models, feature extraction and feature classification are merged to perform simultaneously in a purely data-driven manner in a single framework, which greatly alleviates the need for manual feature extraction. In recent years, more and more researchers have started to explore the application of deep learning in EEG signal recognition, and many achievements have been achieved. Reference [9] designed and trained various deep convolutional neural networks (CNNs) with different architectures to decode task-related information from the raw EEG signals. By improving the structure of CNNs, at least as good performance as Filter Bank Common Spatial Pattern (FBCSP) algorithm widely used was achieved. In Reference [10], a 4-layer compact convolutional neural network, EEGNet, was designed to classify EEG signals from four different BCI paradigms (P300, error-related negative (ERN), motion-related cortical potential (MRCP), and sensori motor rhythm (SMR)). The experimental results showed that EEGNet achieved good scaling across paradigms with sufficient robustness. This study is of great value for EEG signal recognition cross-tasks and cross-subjects. Reference [11] proposed a deep learning method based on a restricted boltzmann machine (RBM) for motor imagery EEG signals classification. Three RBM models are trained by obtaining the frequency domain representation of EEG signals through fast fourier transform (FFT) and wavelet packet decomposition (WPD). These RBMs are then stacked with additional output layers to form a four-layer neural network called frequency depth belief network (FDBN). In Reference [12, 13], long short-term memory (LSTM) was used to process MI-EEG signals, and the accuracy was 77.30% and 82.75%, respectively. Reference [14] proposed an MI-EEG signal recognition algorithm combining bidirectional LSTM (BiLSTM) and graph convolutional neural network (GCN), using BiLSTM to extract relevant features from original EEG signals, and the connected GCN by cooperating with the topological structure of features improves decoding performance. Reference [15] proposed a classification framework for MI-EEG signals that combines a convolutional neural network (CNN) architecture with a variational autoencoder (VAE) for classification. Reference [16] introduced two deep learning-based frameworks with novel spatiotemporal preserving representations of raw EEG streams to precisely identify human intentions. The two frameworks consist of both convolutional and recurrent neural networks, effectively exploring the preserved spatial and temporal information in either a cascade or a parallel manner. Reference [17] proposed a subject-independent framework based on deep convolutional neural networks. In this framework, it formulated the discriminative feature representation as a combination of the spectral-spatial input embedding the diversity of the EEG signals, as well as a feature representation learned from the CNN through a fusion technique that integrates a variety of discriminative brain signal patterns. Reference [18] proposed a novel multimodal machine learning (ML) based approach is proposed to integrate EEG engineered features for automatic classification of brain states. Reference [19] proposed a novel method based on conditional empirical mode decomposition (CEMD) and a one-dimensional multiscale convolutional neural network (1DMSCNN) is proposed to recognize motor imagery EEG signals. This method can provide a stimulus to the development of human-robot interaction.

In summary, deep learning-based methods have unique advantages over traditional methods. Deep learning can automatically extract features applied to classification from original signals and improve classification accuracy. Feature extraction and feature classification are combined in the one frame to avoid information loss caused by different objective functions of the two stages when feature extraction and feature classification are performed step by step. On the other hand, deep learning expands the scope of applications across paradigms and subjects. However, its application still faces many challenges, and the existing EEG signal recognition algorithms still cannot meet the needs of complex application scenarios of brain-computer interfaces. In addition, it makes accurate recognition more difficult due to the low signal-to-noise ratio and non-stationary and nonlinear characteristics of EEG signal. The limitation of the deep learning algorithm itself is also a bottleneck restricting the development of brain-computer interfaces. The current deep learning-based MI-BCI research mainly faces the following challenges:

- The EEG signal is very weak, with the amplitude usually less than 200 *μ*V. Motor imagery EEG signal is the spontaneous random signal with nonstationary randomness in time, and slight changes in the external environment or body state may cause unpredictable effects [20]. In addition, the signal is easily mixed with noise signals from the external environment and from such as electrocardiogram (ECG), respiration, electrooculogram (EOG), and EMG artifacts caused by human activities, resulting in a very low signal-to-noise ratio of EEG.
- There is still a lack of large samples of labeled EEG datasets, which makes the effectiveness of existing deep learning algorithms not fully reflected. The effectiveness of deep learning greatly depends on high-quality labeled data. However, in existing studies, especially in clinical studies, well-labeled and accurate EEG data are still scarce, and the sample size is small. Moreover, it is unrealistic to obtain data sets that can train complex neural network models from a single subject.
- There is too much individual variability across subjects. Many deep learning algorithms that perform well on a single dataset do not perform as well as expected on other datasets or in other online systems. So far, no one or several algorithms have performed well on all datasets, subjects, or data from the same subject at different time periods.

Based on this, a DSC-ConvLSTM model based on attention mechanism is proposed to extract the effective features of motor imagery EEG signals for classification. To address challenge i, for EEG data with a low signal-to-noise ratio, we designed the model architecture according to the characteristics of multichannel motor imagery EEG signals so that it could effectively extract significant features of EEG signals and improve the accuracy of EEG classification. To address challenge ii, for the small sample size of the well-labeled EEG dataset, we use sliding windows to enhance data before the training network, which is equivalent to increasing the training sample size. To address challenge iii, for the problem of cross-subject variation, we take this into account in the detailed design of the model. In terms of data samples, the data augmentation using the sliding window not only increases the sample size but also makes the network no longer extract global features of the whole trial, making the model diminish learning the individualized characteristics of every subjects' EEG data. Also, in terms of model design, as EEG signals have complex spatio-temporal characteristics, we use a convolutional neural network (CNN) to extract spatial frequency information of EEG, in which depthwise separable convolution (DSC) structure is introduced. On the one hand, the general characteristic of DSC is that it can reduce the number of parameters. On the other hand, for EEG decoding, DSC uses depthwise convolution to extract individual feature mappings in time and pointwise convolution to optimize the combination of feature mappings, and different feature maps can represent the information data of different time scales of EEG signals, which can explicitly decouple the relationships within and across feature mappings and alleviate the problem of individual differences to a certain extent. In view of the fact that EEG data is a kind of temporal data, in addition to using CNN to capture spatial features of EEG signals sequences, we will also use bidirectional Convolution Long Short Time Memory (BiConvLSTM) based on the attention mechanism capturing the temporal evolution of EEG signals sequence. All architectures of the model are designed for the characteristics of multichannel motor imagery EEG signals. In order to verify the validity of the proposed model, we compare it with other novel methods on the decoding accuracies, such as filter bank common spatial patterns (FBCSP) in Reference [8], Deep ConvNets, and Shallow ConvNets in Reference [9], EEGNet-4,2, EEGNet-8,2 in Reference [10]. By analyzing the decoding performance of different models in both the BCI Competition IV Dataset 2a and the High Gamma Dataset. The experimental results show that the DSC-ConvLSTM model based on the attention mechanism proposed in this paper can effectively decode MI-EEG signals with certain stability and robustness, which can effectively mitigate the intertrial and intersubject differences.

The paper is structured as follows: Section 1 is the introduction, Section 2 introduces the datasets we use and the details of our proposed model, Section 3 shows the experimental results and the discussion, and Section 4 is the conclusion.

## 2. Materials and Methods

### 2.1. Data Description and Processing

The motor imagery BCI (MI-BCI) system is mainly input by EEG signals caused by the rhythmic changes of the cerebral cortex stimulated by motor imagery. The physiological principle of motor imagery EEG is based on event-related synchronization (ERS)/event-related desynchronization (ERD) [21]. When people engage in some specific exercise or mental activity, the coherent area of the cerebral cortex is activated, and thus the metabolism and blood flow in this area are accelerated, resulting in a decrease in the amplitude of the frequency spectrum oscillation of brain waves in some frequency bands, corresponding to a decline in the frequency band energy. This physiological phenomenon is known as ERD. In another situation, when the brain is in a quiet or inert state, specific frequency bands show a significant increase in amplitude, which corresponds to an increase in band energy known as ERS. The ERS/ERD phenomenon generally occurs in the frequency range of 8–12 Hz and 18–26 Hz [22], where most of the sensory-motor cortex potential generated by adults is located. This is the physiological basis for the classification of motor imagery EEG signals. The datasets used in this experiment are BCI Competition IV Dataset 2a [23] and High Gamma Dataset [9].

#### 2.1.1. Dataset 1: BCI Competition IV Dataset 2a

The BCI competition IV Dataset 2a (BCIC IV 2a) consisted of EEG data from nine subjects, and its experimental paradigm consisted of four different motor imagery tasks: left hand, right hand, feet, and tongue. Each subject completed two sessions on different days, and each session consisted of six runs with a short break between each run. Each run consisted of 48 trials (12 for each task). Thus, there are a total of 288 trials per session. EEG signals were recorded by 22 Ag/Al electrodes at a sampling frequency of 250 Hz, bandpass filtered between 0.5 Hz and 100 Hz, and line noise suppressed by a 50 Hz trap filter.

#### 2.1.2. Dataset 2: High Gamma Dataset

Compared with Dataset 1, the High Gamma Dataset (HGD) is a larger dataset with a total of 20 subjects. A trial per subject was divided into 13 runs, for a total of approximately 1000 (992 ± 135.3) trials. The four types of motor imagery tasks were left hand, right hand, feet, and rest (the rest had no specific movement but had the same visual cues as other tasks in the paradigm design). Except for the last two runs, the training set consisted of approximately 880 trials and the test set consisted of approximately 160 trials. The data were recorded by 128 electrodes at a sampling frequency of 5 kHz. And the noninvasive detection of high-frequency EEG components in the sensorimotor cortex due to ERS/ERD phenomenon was optimized [24]. In the experiments, the HGD dataset was resampled to 250 Hz to ensure that it was identical to the BCIC IV 2a dataset so that the network parameter settings could be adapted to both datasets.

**Figure 1:**
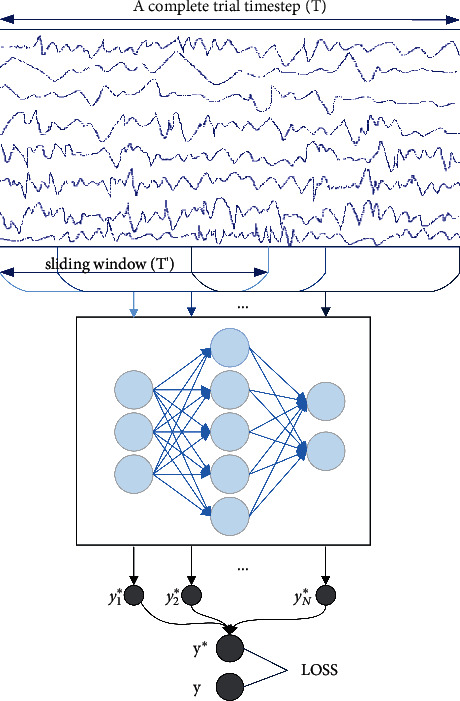
Sliding windows for data augmentation. *T* denotes the time step of the whole trial, *T*' denotes the time length of the sliding window, *y*_1_^*∗*^, *y*_2_^*∗*^,…, *y*_*N*_^*∗*^ is the prediction value obtained for each example of the sliding window, and the final loss is the difference between the mean of all predictions *y*^*∗*^ and the target *y*.

Due to the small sample size of the EEG dataset, we use the sliding window for data augmentation prior to network training instead of using the entire trial directly as input to the network. This allows the trials to be divided into multiple smaller trials, increasing training samples. The schematic diagram of data augmentation is shown in Figure 1. In this study, the time range of samples we took was from −0.5 s before the start of each trial to 4s after the start of each trial which achieved the best results we had ever experimented. For each sliding window, the samples within each sliding window segment are used as a new example. For example, given an original trial *X*^*i*^=*R*^*C*·*T*^ of subject *i*, where *C* denotes the number of channels and *T* denotes the time step. The time length of the sliding window is *T*′, then after multiple window slides, each trial will get multiple new examples *X*^*i*′^=*R*^*C*·*T*′^. The time step of each sliding window is 2 s, the start point of the first sliding window is 0.5 s before the start of each trial, and the start point of the last sliding window is 2 s after the start of each trial, that is, the end point is 4 s after the start of each trial. Therefore, the whole range is from 0.5 s before the start of each trial to 4 s after the start, which is the same as our original setting. We believe that it has a higher recognition accuracy when windows are in the vicinity of 0.5 s to 2.5 s after the start of each trial. Because the subjects are most focused, just starting to concentrate, and do not appear to have motor imagery fatigue. According to the above Settings, the starting point of the sliding window ranges from −0.5 s to 2 s, a total of 2.5 s. The signal sampling frequency was 250 Hz, and 625 new samples were generated for each trial. That is, the number of our training samples increased by 625 times. These new examples are trained separately by the network, and the average of the predictions will be used as the final prediction of the overall trial, and the loss of the overall trial is calculated by comparing with the target value. This data preprocessing approach not only increases the number of training samples but also make the network no longer extract global features and individualized features of every trial.

### 2.2. Overall Network Structure

**Figure 2:**
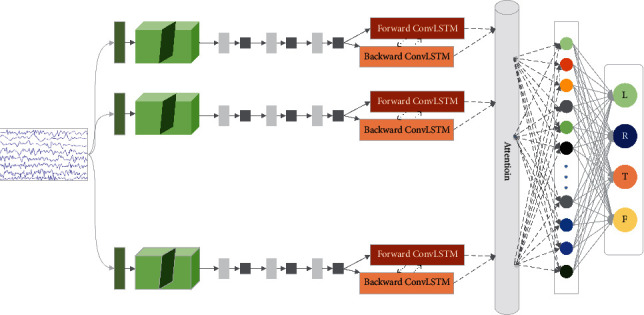
EEG decoding overall network structure. The input signal form is *X*^*i*^=*R*^CT^, where *C* denotes the number of channels and *T* denotes the time step. To simplify the network structure diagram, the pooling layer of each block is not represented in the diagram.

Because EEG signals are nonstationary and stochastic, and the sample size is small but contains abundant information. The signal acquisition process is susceptible to environmental and individual variability, so it is difficult to effectively guarantee the robustness of the classification system. In this paper, a DSC-ConvLSTM network structure is proposed according to the characteristics of EEG signals, as Figure 2 shows. The final convolutional classification layer further fuses the feature information extracted by DSC-ConvLSTM and maps the extracted feature matrix into the sample marker space to achieve end-to-end classification and recognition of EEG signals.

EEG signals have complex temporal and spatial characteristics, so the overall model is based on the basic structure of CNN-LSTM. The deep convolutional neural network can learn the spatial frequency information of EEG signals by continuous training. In addition, considering the multichannel characteristics of EEG signals, the deep separable convolutional structure (DSC) is used in the CNN module. The CNN module ignores the information of the temporal domain, while the LSTM module can capture the temporal evolution of EEG signal sequences. The gate mechanism of LSTM enables the trained model to adapt to long-term dependence, which is conducive to the serialization feature extraction of EEG signals. In addition, considering the contextual temporal characteristics and underlying spatial features of EEG signals, we improve the single LSTM unit and propose a new ConvLSTM structure to classify EEG signals. ConvLSTM changes the existing structure of LSTM unit, where the state-to-state conversion of classical LSTM adopts a fully connected form, while ConvLSTM adopts a convolutional form. In addition, the attention mechanism is introduced to select the information which is more critical to the current task objective from the numerous information to improve the decoding accuracy.

### 2.3. Depthwise Separable Convolution

The DSC module network structure and parameter settings are shown in Table 1, which consists of 4 blocks. The first block is special, designed to better process EEG signals as input. The other three blocks are all standard convolution-max-pooling.

In Block1, the convolution module is divided into two layers: time-domain convolution and spatial filtering. The EEG signal input into the network is a two-dimensional array, whose width is the number of time steps and its length is the number of electrode channels. And unlike images that have three RGB color channels, each electrode of EEG signals is one channel. In the first layer time-domain convolution is designed as F1 2D convolution filters with kernel size of (1, 64), and this layer outputs F1 feature maps containing EEG signals with different bandpass frequencies. Then in the second layer spatial filter uses a Depthwise Convolution, which is designed as D*∗*F1 2D Convolution filters with the kernel size (*C*, 1) to extract the spatial domain features of EEG signals in the electrode channel dimension. The advantage of Depthwise Convolution is that it reduces the number of network training parameters. Different from the conventional convolution operation, one convolution kernel of Depthwise Convolution is responsible for one channel, and one channel is only convolved by one convolution kernel, so there are as many filters as there are channels. After the new channel feature maps are obtained, the standard 1*∗*1 cross-channel convolution operation is then performed on this batch of new channel feature maps. Depthwise Convolution operation reduces the number of parameters compared to a standard convolution operation and has better results because it learns each channel of the EEG signal (each channel corresponds to a different filter instead of all channels corresponding to the same filter). The depth parameter *D* controls the number of spatial filters that need to be learned for each feature mapping, and here we set *D* to 1. This two-layer convolution pattern is somewhat inspired by the Filter-Bank Common Spatial Pattern (FBCSP) algorithm [25].

In Block2-Block4, there are basically similar modules. In Block2, for example, the separable convolution is used, whose core idea is to decompose a complete convolution operation into two steps: Depthwise Convolution and Pointwise Convolution [26]. Here, the separable convolution consists of F2 Depthwise Convolution with kernel size (1, 16) and D*∗*F2 Pointwise Convolution with kernel size (1, 1). Its main advantages: One is to reduce the number of network parameters; the other is to decouple the relationship within and across feature mappings explicitly. Depthwise Convolution is used to summarize each feature map, respectively, and finally, Pointwise Convolution is used to optimize and combine the output. This is very suitable for EEG signal classification characteristics, where different feature mappings can represent data from different time scales of EEG signals.

The activation function is exponential linear unit (ELU) [27], which is more sensitive to feature types to improve the classification performance compared to Square and ReLU that generate average power. Before the ELU unit, batch normalization (BN) is applied on each dimension of feature mapping and the entire model is regularized using dropout [28], where the dorpout rate is set to 0.5 in the validation of the dataset between individual subjects to prevent overfitting of the network on small data samples. At the same time, the linear transformation is also forced to separate into a combination of time-domain convolution and spatial filtering, implicitly regularizing the entire model. The pooling method is chosen as max-pooling, because for the most common image applications, max-pooling preserves the texture features, and mean-pooling preserves the overall data features. At the same time, different features of the EEG signal may have different manifestations in high-frequency and low-frequency components. Compared to mean-pooling, max-pooling extracts the features with the largest and strongest responses and inputs them to the next module. Only the last block uses mean-pooling to ensure the integrity of EEG data information. Softmax is used for the final classification layer.

### 2.4. ConvLSTM with Attention

**Figure 3:**
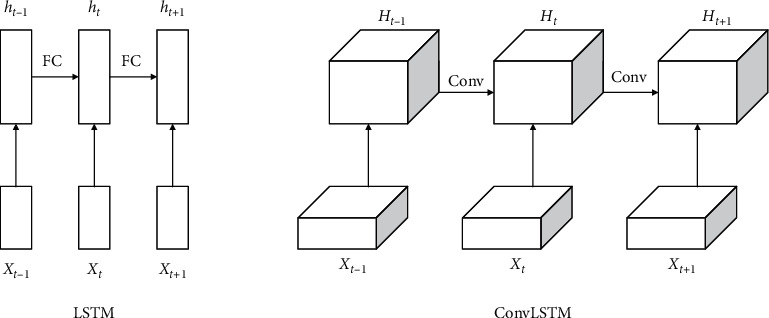
Structural differences between LSTM and ConvLSTM.

**Figure 4:**
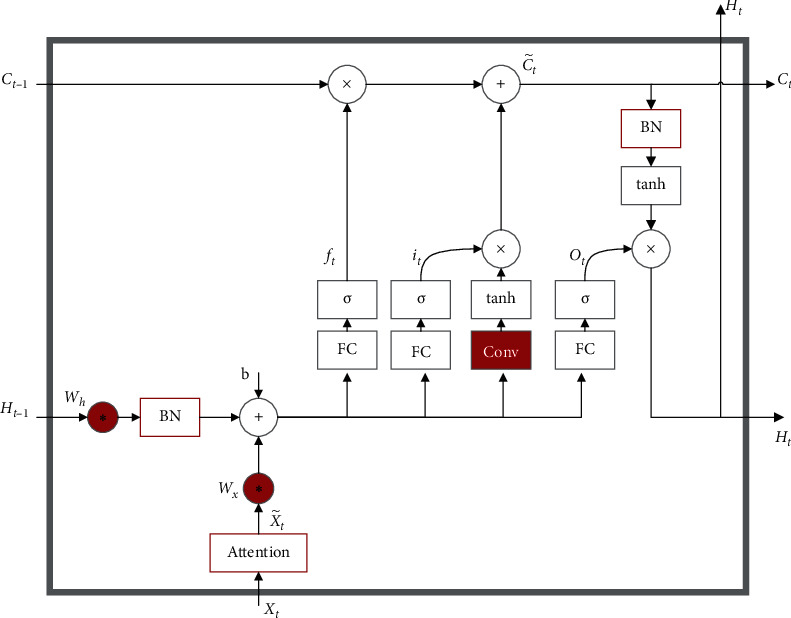
ConvLSTM network structure.

LSTM is an improvement of recurrent neural network (RNN), which retains the ability of RNN to model sequence data accurately. In order to solve the problem of gradient disappearance or explosion during the training process of RNN, LSTM added memory unit C, input gate I, forgetting gate F, and output gate O, the input gate obtains new input from the outside and processes new data. The forgetting gate determines when to selectively forget a particular output result, so as to select the best time lag for the input sequence. The output gate computes all the results and generates the output for the LSTM unit. The main difference between ConvLSTM and LSTM is that standard LSTM adopts full connection form in input-to-state and state-to-state connection, and decoding does not take into account spatial structure characteristics, while ConvLSTM adopts the form of convolution. The difference in details between the two is shown in Figure 3. For the ConvLSTM structure, all the inputs input *x*_*t*_, cell output *C*_*t*_, hidden layer state *H*_*t*_, and *f*_*t*_, *i*_*t*_, *o*_*t*_ are 3D Tensor, where two dimensions are spatial features. However, the input dimension of the traditional LSTM module is one-dimensional, which can be regarded as the two dimensions of spatial features are 1, which is not applicable to spatial sequence data. Therefore, the convolution form of input and state connection can fully extract the spatial features of EEG. However, since the previous DSC module has already focused on significant spatial regions with valuable spatiotemporal information, we expect the ConvLSTM module to only focus on spatiotemporal feature fusion. To further optimize the model, global mean pooling is performed for input features and hidden states to reduce the spatial dimensionality. In addition, we did several experiments: (a) removing the convolutional structure in the three gates, and the convolutional structure was only introduced into the input-to-state transformation; (b) leaving the convolutional structure in the three gates; (c) leaving the convolutional structure only in the input gate/forgetting gate/output gate. The result is optimal in all experiments when the convolution structure is only introduced into the input-to-state transition, while the gate still remains the original fully connected mechanism. The added convolutional structure enables LSTM to perform spatiotemporal feature fusion, while the gate mechanism retains its advantage in long-term temporal fusion. The internal network structure of ConvLSTM is shown in Figure 4. Equations (1)–(7) show the process of ConvLSTM.where *σ* is the sigmoid layer, the symbol “*∗*” denotes the convolution operator, and “·” denotes the Hadamard product. The process is as follows: The first step is to decide what information to discard from the cell states, which is done by the forget gate. As shown in equation (3), the gate reads *h*_*t*−1_ and *x*_*t*_, and outputs a value between 0 and 1 to each number in the cell state. 1 means “keep completely” and 0 means “discard completely.” The next step is to determine what new information is stored in the cell state. As shown in equations (4) and (5), the sigmoid layer, called the “input gate layer,” determines what values we will update and the updated values are *i*_*t*_. Then, a tanh layer creates a new vector of candidate values ${\tilde{C}}_{t}$ that will be added to the state. Finally, we need to determine what value to output. This output will be based on the state of our cell. As shown in equation (6), a sigmoid layer is run to determine which part of the cell state will be output. As shown in equation (7), we take the cell state and process it through tanh (to get a value between −1 and 1) and multiply it with the output of the sigmoid gate, and we end up outputting only the part that we determined to be output.


(1) $$\begin{array}{c} {\tilde{X}}_{t}=\text{GlobaL Mean Pooling}\left(X_{t}\right), \end{array}$$

(2) $$\begin{array}{c} {\tilde{\text{H}}}_{t-1}=\text{GlobaL Mean Pooling}\left(H_{t-1}\right), \end{array}$$

(3) $$\begin{array}{c} f_{t}=\sigma \left(W_{\text{xf}}{\tilde{X}}_{t}+W_{\text{hf}}{\tilde{H}}_{t-1}+b_{f}\right), \end{array}$$

(4) $$\begin{array}{c} i_{t}=\sigma \left(W_{\text{xi}}{\tilde{X}}_{t}+W_{\text{hi}}{\tilde{H}}_{t-1}+b_{i}\right), \end{array}$$

(5) $$\begin{array}{c} {\tilde{C}}_{t}=f_{t}\cdot C_{t-1}+i_{t}\cdot \text{tanh}\left(W_{\text{xc}}*{\tilde{X}}_{t}+W_{\text{hc}}*{\tilde{H}}_{t-1}+b_{c}\right), \end{array}$$

(6) $$\begin{array}{c} o_{t}=\sigma \left(W_{\text{xo}}{\tilde{X}}_{t}+W_{\text{ho}}{\tilde{H}}_{t-1}+b_{o}\right), \end{array}$$

(7) $$\begin{array}{c} H_{t}=o_{t}\cdot \text{tanh}\left({\tilde{C}}_{t}\right). \end{array}$$

**Figure 5:**
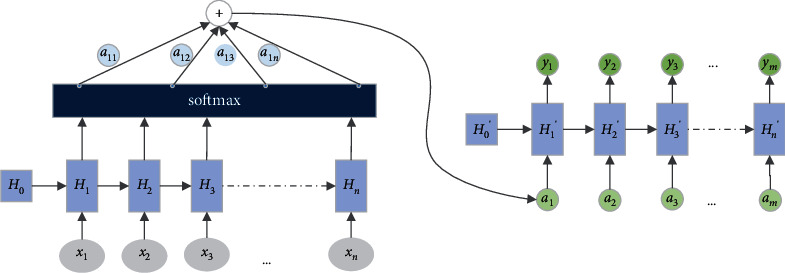
Structure diagram of attention mechanism.

In addition, the attention mechanism is introduced into the model. The structure of the attention mechanism is shown in Figure 5. The mechanism learns the importance of each element from the sequence and then merges elements according to their importance. In the process of improving the model by using the attention mechanism, we tried to introduce attention mechanism at different points in the network, such as introducing an attention mechanism on the input and improving the forgetting gate/input gate/output gate by utilizing the attention mechanism on the channel. We find that it is better to introduce attention mechanism in the input, and the specific attention mechanism embedded in the three gates does not facilitate feature fusion, but it only brings additional memory and computing consumption. The process of attention mechanism can be described as follows:

(8) $$\begin{array}{c} Z_{t}=\text{tanh}\left(W_{w}*H_{t}+b_{w}\right), \end{array}$$

(9) $$\begin{array}{c} a_{t}=p\left({\text{att}}_{t}H_{t}\right)=\frac{\exp\left(Z_{t}^{\Gamma }Z_{w}\right)}{\sum_{t}\exp\left(Z_{t}^{\Gamma }Z_{w}\right)}, \end{array}$$

(10) $$\begin{array}{c} y_{t}=\sum_{t}a_{t}•H_{t}. \end{array}$$

The output *y*_*t*_ of the attention mechanism module is the weighted sum of *a*_*t*_ for the output *H*_*t*_ of the ConvLSTM module. *Z*_*t*_ is the output of the last classification layer, *a*_*t*_ is the output of the probability distribution of the softmax layer, where *W*_*w*_, *Z*_*w*_ and *b*_*w*_ are the weights and bias of training, and the most significant features are selected from the output of the ConvLSTM module through the weights *a*_*t*_, because *a*_*t*_ represents the contribution to the decoding task.

Alternatively, we use bidirectional ConvLSTM, where two ConvLSTM units with different directions are fused and communicate with the context as a complete module. The bidirectional ConvLSTM consists of two unidirectional ConvLSTM networks stacked forward and backward, where $\overset{⟶}{\text{ConvLSTM}}$denotes the prediction of the forward input signal sequence from the beginning to the end, calculated forward from 1 to *t* to obtain and save the output of the forward hidden layer at each moment and $\overset{\leftarrow }{\text{ConvLSTM}}$ denotes prediction of the backward input signal sequence from the end to the beginning, calculated backward from moment *t* to 1 to obtain and save the output of the backward hidden layer at each moment. The two ConvLSTM units perform information fusion in the hidden layer to predict the input at the current moment *t*. The output is determined jointly by the two ConvLSTM networks. The calculation is performed by error backpropagation in the BiConvLSTM module. The error backpropagation in BiConvLSTM module is similar to the BP algorithm, which continuously searches for better points along the negative gradient direction of parameters to be optimized until model converges. The backpropagation of the error term also includes two directions: One is the backpropagation along time, that is, the error term at each moment is calculated from the current moment *t*; One is to propagate the error term up one layer in BiConvLSTM module. The algorithm process of error back propagation is as follows: Firstly, the output value of each neuron was calculated forward, and there were five variable *f*_*t*_, *i*_*t*_, ${\tilde{C}}_{t}$, *o*_*t*_ and *h*_*t*_. We give the forward propagation formula of ConvLSTM as equations (3)–(7). Then the error term of the neuron is calculated backwards. The error propagates step by step by gradients *δ* of hidden state neurons. The hidden states are *f*_*t*_ and ${\tilde{C}}_{t}$. Here, two gradient variables *δ*_*h*_^(*t*)^ and *δ*_*C*_^(*t*)^ are defined, so the gradient of the final sequence index position is *δ*_*h*_^(Γ)^ and *δ*_*C*_^(*t*)^. The gradient of *δ*_*h*_^(Γ)^ is determined by the output gradient error of this layer, while the reverse gradient error of *δ*_*C*_^(Γ)^ is composed of the gradient error of *δ*_*C*_^(*t* + 1)^ in the previous layer and the gradient error coming back from *δ*_*h*_^(*t*)^ in this layer. Therefore, only *δ*_*C*_^(*t*)^ is used in the backpropagation, and the variable *δ*_*h*_^(*t*)^ is only used to help us calculate at a certain layer and does not participate in the backpropagation. With *δ*_*h*_^(Γ)^ and *δ*_*C*_^(Γ)^, it is easy to calculate the various weight parameters *W*_xf_, *W*_hf_, *W*_xi_, *W*_hi_, *W*_xc_, *W*_hc_, *W*_xo_, *W*_ho_, *b*_*f*_, *b*_*i*_, *b*_*c*_, *b*_*o*_.

## 3. Results and Discussion

**Figure 6:**
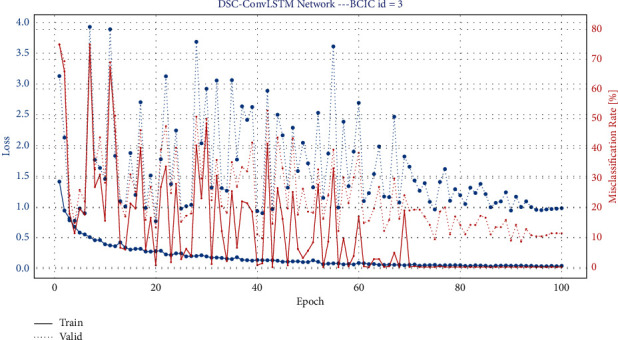
Iteration results_BCIC IV 2a. The red solid line and dashed line indicate the change in accuracy of the training and validation sets during the iterative process, respectively, while the blue solid line and dashed line indicate the change in LOSS of the training and valid sets during the iterative process.

**Figure 7:**
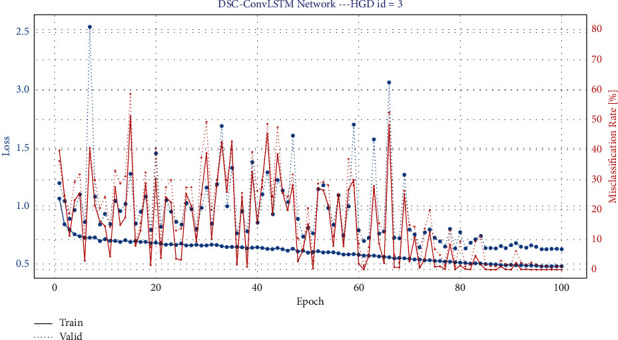
Iteration results_High gamma dataset. The red solid line and dashed line indicate the change in accuracy of the training and validation sets during the iterative process, respectively, while the blue solid line and dashed line indicate the change in LOSS of the training and valid sets during the iterative process.

**Figure 8:**
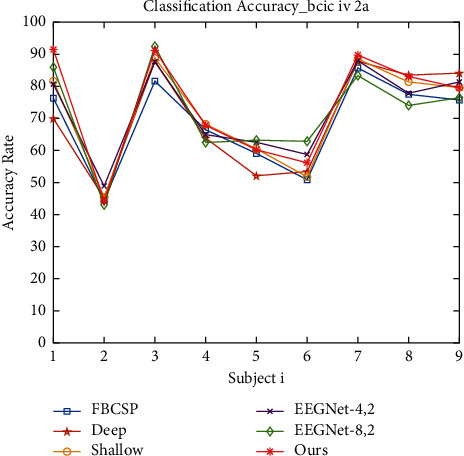
Comparison results of the classification accuracy on BCIC IV 2a dataset.

**Figure 9:**
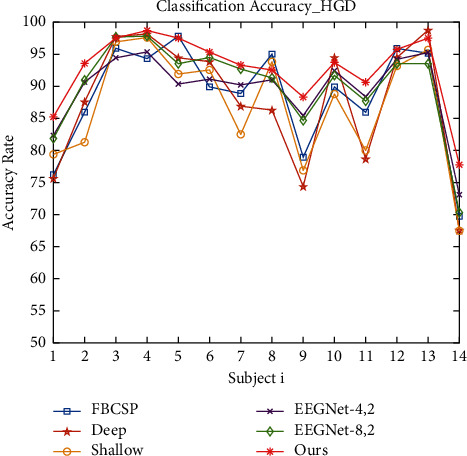
Comparison results of the classification accuracy on the high gamma dataset.

This session mainly introduces the training and testing of the model. The model is trained using the Adam optimizer to make the model converge better. And the model is trained by minimizing the classification cross-entropy loss function. We use 4-Fold cross-validation, where two sets are training sets, one set is a validation set, and the last set is a test set. As in baseline, we use a time window of −0.5 s~4 s for all datasets. The hyperparameters of the model, including batch size, learning rate, convolutional kernel size, etc., were continuously tuned and optimized. Figures 6 and 7 show the iteration curves during the training process, demonstrating the iterative changes in accuracy and LOSS. Accuracy converges of the training set close to 1, and the LOSS is close to 0. It shows that the model achieves good convergence. The computational complexity of our model is shown in Table 2 (It should be noted that the number of parameters in the table is calculated based on the BCIC IV 2a dataset). The classification accuracy results are shown in Tables 3 and 4. A visual comparison of the classification accuracy of our method with other baseline methods is shown in Figures 8 and 9.

As can be seen from Tables 3 and 4, our method achieves better results in decoding accuracy on both datasets. Our model achieves an average classification accuracy of 73.7% and 92.6% on BCIC IV 2a and HGD datasets, respectively. The decoding performance is improved on both datasets, which demonstrates the generality of the method we proposed. Moreover, the results are validated by 4-Fold cross-validation, which proves the model in this paper reduces the effect of EEG signal variability among different subjects to a certain extent and improves the overall robustness and stability of the model, which is relevant for the application of motor imagery BCI devices.

To further understand the importance of each element of the model for the deep learning structure, we have done an ablation study, as shown in Table 5. The most initial structure is the CNN-LSTM model. The accuracy of the original model in the two datasets is 58.6% and 78.9%, respectively. The design structure in the table is the improvement of each step on the initial structure, and the improvement process is from top to bottom. It clearly shows the purpose of our improved structure and its effect on improving accuracy.

## 4. Conclusion

To extract and classify motor imagery EEG signal features more accurately, the DSC-ConvLSTM model based on the attention mechanism is proposed in this paper. Due to the small sample size of existing EEG datasets, but the effectiveness of deep learning greatly still depends on high-quality labeled data. In this paper, a sliding window is firstly used for data enhancement, which not only meets the requirements of deep learning models for large sample datasets but also makes the network no longer extract global characteristics of the whole trial, making the model diminish learning the individualized characteristics of every subjects' EEG data, which can effectively eliminate the influence of individual specificity and instability of EEG signals on model training. In the aspect of model construction, the convolutional neural network is used to extract the temporal and spatial frequency features of EEG signals, in which a deep separable convolutional structure is used, which not only reduces the number of network parameters and training time but also facilitates the direct feature extraction of EEG signals. Then the bidirectional ConvLSTM based on the attention mechanism is used to extract the temporal characteristics of EEG signals. ConvLSTM improves the existing LSTM cell structure, and the state-to-state conversion of ConvLSTM adopts the form of convolution. And considering the compromise between the number of parameters and performance, global mean pooling is performed for input features and hidden states to reduce the spatial dimension, and convolution is only introduced into the input-to-state transitions for spatiotemporal feature fusion, while the weights for other unnecessary transitions between other gates do not take the convolutional form. In addition, we introduce an attention mechanism only in the input to improve the classification accuracy. The results show that our model achieves better results on different datasets and makes further progress in effectiveness and robustness, which can provide finer granularity, more content, more stable and real-time system for the corresponding brain-computer interface domain, thus extending the brain-computer interface functions and improving the efficiency of brain-computer interface systems.

In the near future, we will continue keeping the focus on the research on improving the classification accuracy of EEG signals. As for the individual difference of the same network model, we will try to learn more about the algorithms of feature extraction and feature classification to improve the classification accuracy across different subjects. We believe that transfer learning can be a breakthrough to solve this kind of problem.

## Figures and Tables

**Table 1:** Network structure.

Blocks	Layer	Filters	Output	Options
1	Input		[C, T]	
	Reshape		[1, C, T]	
	Conv_time	F1	[F1, C, T]	kernel_size = (1, 64), stride = (1, 1), padding = (0, F1*∗*2)
	BatchNorm		[F1, C, T]	num_features = F1, eps = 0.001, momentum = 0.01
	Conv_spat	D*∗*F1	[D*∗*F1, 1, T]	kernel_size = (C, 1), stride=(1, 1),groups = F1
	BatchNorm		[D*∗*F1, 1, T]	num_features = D*∗*F1, eps = 0.001, momentum = 0.01
	Activation		[D*∗*F1, 1, T]	ELU
	MaxPool2D		[D*∗*F1, 1, T//3]	kernel_size = (1, 3),stride = (1, 3)
	Dropout		[D*∗*F1, 1, T//3]	*p*=0.25

2	DepthwiseConv2D	F2	[F2, 1, T//3]	kernel_size = (1, 16), stride = (1, 1), groups = F2
	PointwiseConv2D	D*∗*F2	[D*∗*F2, 1, T//3]	kernel_size = (1, 1), stride = (1, 1)
	BatchNorm		[D*∗*F2, 1, T//3]	num_features = D*∗*F2, eps = 0.001, momentum = 0.01
	Activation		[D*∗*F2, 1, T//3]	ELU
	MaxPool2D		[D*∗*F2, 1, T//9]	kernel_size = (1, 3), stride = (1, 3)
	Dropout			*p*=0.25

3	DepthwiseConv2D	F3	[F3, 1, T//9]	kernel_size = (1, 16), stride = (1, 1), groups = F3
	PointwiseConv2D	D*∗*F3	[D*∗*F3, 1, T//9]	kernel_size = (1, 1), stride = (1, 1)
	BatchNorm		[D*∗*F3, 1, T//9]	num_features = D*∗*F3, eps = 0.001, momentum = 0.01
	Activate		[D*∗*F3, 1, T//9]	ELU
	MaxPool2D		[D*∗*F3, 1, T//27]	kernel_size = (1, 3), stride=(1, 3)
	Dropout		[D*∗*F3, 1, T//27]	*p*=0.25

4	SeparableConv2D	F4	[F4, 1, T//27]	kernel_size = (1, 16), stride = (1, 1), groups = F4
	PointwiseConv2D	D*∗*F4	[D*∗*F4, 1, T//27]	kernel_size = (1, 1), stride = (1, 1)
	BatchNorm		[D*∗*F4, 1, T//27]	num_features = D*∗*F4, eps = 0.001, momentum = 0.01
	Activation		[D*∗*F4 ,1, T//27]	ELU
	MeanPool2D		[D*∗*F4, 1, T//81]	kernel_size = (1, 3), stride = (1, 3)
	Dropout		[D*∗*F4, 1, T//81]	*p*=0.25

**Table 2:** Number of trainable parameters per model.

	DeepConvNet	ShallowConvNet	EEGNet-4,2	EEGNet-8,2	Ours
Number of parameters	152219	40644	796	1716	17972

**Table 3:** Within-subject classification accuracy_bcic iv 2a.

Author	Algorithm	Accuracy (%)									
		A01	A02	A03	A04	A05	A06	A07	A08	A09	Mean
Sakhavi S	FBCSP	76.11	44.33	81.52	66.30	58.96	50.75	85.69	77.35	75.63	68.0
R. T. Schirr	Deep ConvNet	69.75	45.44	87.46	63.85	52.04	53.39	87.46	83.46	83.99	69.6
	Shallow ConvNet	81.60	45.49	88.54	68.06	60.42	51.74	88.54	81.25	79.51	71.7

V. J. Lawhern	EEGNet-4, 2	80.56	48.96	87.50	64.93	62.50	58.68	87.85	77.78	81.25	72.2
	EEGNet-8, 2	85.76	43.06	92.36	62.50	63.19	62.85	83.33	73.96	76.39	72.5

Ours	Ours	91.32	44.10	90.97	67.71	60.07	56.25	89.58	82.99	79.51	73.7

**Table 4:** Within-subject classification accuracy_HGD.

Algorithm	Accuracy (%)														Mean
	A01	A02	A03	A04	A05	A06	A07	A08	A09	A10	A11	A12	A13	A14	
FBCSP	76.12	85.92	95.79	94.22	97.73	89.89	88.87	94.90	78.90	89.87	85.87	95.78	95.12	69.70	88.5
Deep ConvNet	75.62	87.50	97.50	98.12	94.37	93.75	86.88	86.25	74.38	94.37	78.56	94.37	98.75	67.50	88.1
Shallow ConvNet	79.37	81.25	96.88	97.50	91.87	92.50	82.50	93.75	76.88	88.75	80.00	93.13	95.63	67.50	87.0
EEGNet-4, 2	82.32	90.63	94.36	95.31	90.34	91.10	90.13	90.99	85.36	92.34	88.33	94.18	95.21	73.10	89.6
EEGNet-8, 2	81.81	90.86	97.63	97.67	93.48	94.46	92.66	91.29	84.66	91.64	87.63	93.48	93.51	70.40	90.1
Ours	85.25	93.55	97.46	98.68	97.47	95.27	93.28	92.44	88.20	93.66	90.65	95.67	97.38	77.79	92.6

**Table 5:** Evaluated deep learning structure.

Design structure	Our choice	Aim	Average accuracy	
			bciv iv 2a (%)	HGD (%)
Convolution in first layer	Splitted convolution	The first layer of convolution is divided into time-domain convolution and spatial filtering, which can better process the input of EEG signal and improve the classification accuracy.	60.2	82.3
ConvNet	Separable convolution	One is to reduce the number of network parameters and improve the training speed; the other is to show the relationships within and across decoupled feature maps	60.8	82.9
LSTM	BiConvLSTM	Improves LSTM's disadvantage of extracting only temporal features of EEG signals and enables it to extract spatial features of EEG signals	65.3	84.6
Data processing	Sliding window	It not only increases the number of training samples but also fully extracts the differential features and global features of all EEG data	73.7	92.6

## Data Availability

BCI Competition IV 2a data set used to support the findings of this study is available at https://www.bbci.de/competition/iv/. High-gamma data set used to support the findings of this study is available at https://web.gin.g-node.org/robintibor/high-gamma-dataset/.
